# Widely used bandit tasks elicit diverging belief updating phenotypes in healthy adults

**DOI:** 10.21203/rs.3.rs-9439488/v1

**Published:** 2026-05-13

**Authors:** Amber E. Kiely, Ember Zhang, Justice Simonetti, Anusha Phadnis, Shuting Yang, Laura A. Berner, Andrew H. Smith, Vincenzo G. Fiore

**Affiliations:** 1 Center for Computational Psychiatry, Department of Psychiatry, Icahn School of Medicine at Mount Sinai, New York, NY, USA; 2 Department of Psychiatry, New York University Grossman School of Medicine, New York, NY, USA; 3 Nathan S. Kline Institute for Psychiatric Research, New York State Office of Mental Health

## Abstract

Adaptive decision-making requires dynamic arbitration between internal beliefs and incoming evidence, yet the role that environment structure plays in shaping these dynamics has received little systematic attention. We used three inductive reasoning and three bandit tasks to investigate these trial-by-trial dynamics in N=120 healthy adults. Using a novel computational model, we found that, in a two-option bandit task with positive valence, as well as in a three-option bandit task with negative valence, a significant number of participants were characterized by suboptimal arbitrations, reflecting increased reliance on priors under high uncertainty and increased reliance on evidence under high confidence. These findings demonstrate that design elements in widely used tasks can bias belief updating dynamics in healthy individuals leading to population-level suboptimal behaviors. These biases may have been overlooked in the past due to simplifications in the characterization of belief updating dynamics, potentially affecting analysis and comparisons with clinical cohorts.

## INTRODUCTION

Adaptive decision-making requires a dynamic arbitration between new sensory information and prior beliefs, formed from past experience, to guide the context-appropriate selection of actions most likely to achieve desired outcomes in uncertain environments^1–4^. Agents update their internal priors in light of new evidence at a pace that is typically determined by three factors: the precision of one’s priors (i.e., confidence in existing beliefs), the magnitude and direction of the prediction error (i.e., the discrepancy between expected and actual outcomes), and the estimated uncertainty in the environment (i.e., how likely an event is, given a particular hypothesis)^5–7^. These factors can override one another. For instance, a large prediction error might typically call for a fast belief update, but in a highly stochastic environment, new information may be better dismissed as noise, favoring a slower update rate. Conversely, a small prediction error might typically be ignored, but it can prompt a meaningful revision of beliefs if a true change point in the environment is detected (e.g., an unambiguous change in action-outcome associations). Finally, strong confidence in one’s priors can lead to ignoring or underweighting new evidence, regardless of prediction error size or environmental volatility^8–10^.

To capture this interplay between sensory information and internal beliefs, reinforcement learning models formalize updating through reward prediction errors and learning rates, while Bayesian models describe inference as the integration of prior beliefs with new evidence. Both frameworks have advanced our understanding of adaptive decision-making and its disruption in psychiatric illness^11,12^, as belief updating (in Bayesian terms) and learning (in reinforcement learning terms) have become a central focus in cognitive neuroscience, behavioral economics and computational psychiatry^10,13–15^. Abnormalities in these processes have been identified across psychiatric disorders, often described in terms of faster, slower or generally impaired, suboptimal or maladaptive learning or prior updating, relative to healthy controls^16,17^. For instance, slowed belief updating has been reported in eating disorders^18^, hallucinations^19,20^, depression^21^ and anxiety^22–24^, where rigid priors maladaptively bias inferences despite contradictory evidence. Conversely, faster updating has been linked to psychosis, where beliefs shift rapidly in response to noisy evidence^25,26^, and to OCD, where hypersensitivity to momentary feedback (particularly negative feedback) can drive excessive reliance on recent observations despite previously accumulated knowledge about the environment^26–28^.

These studies are often characterized by two important limitations. First, they assume that computational processes and deficits generalize uniformly across comparable task environments^29^, for example, that a reduced learning rate or pace of belief updating characterizes a disorder across all bandit tasks. Second, the models employed frequently sacrifice versatility for simplicity and ease of interpretability, limiting the ability to detect meaningful individual or phenotypic differences in computational profiles^30–32^. Together, these limitations may account for apparently contradictory patterns that have been observed within the same disorder. For example, OCD has been characterized both as reflecting excessive sensitivity to momentary feedback and as rooted in rigid, inflexible priors thought to underlie perseverative behaviors^33,34^. Recent work has challenged this inflexibility account entirely^35^, suggesting that the apparent contradictions may reflect something more nuanced than a simple fast-versus-slow dichotomy. While such contradictions are often attributed to clinical heterogeneity or differences in modeling approach^29,36^, an underexplored possibility is that they partly reflect differences in the task environments used to elicit behavior; studies within the same disorder often employ tasks differing in choice complexity, feedback structure, and outcome valence^37,38^, contextual factors that may themselves shape the dynamics under investigation^39^.

Beyond task design, the computational models used to characterize belief updating introduce their own constraints. In their simplest forms, they typically impose subject-specific and fixed updating parameters, assuming for instance a stable learning rate across all trials in a task environment^40,41^ or fixed action-outcome likelihood estimations. This means that these simplified models cannot capture complex and evolving choice dynamics, and may fail to identify nuances in adaptive behaviors that may be specific to task environments or population subgroups^42^. Critically, the very quantities these models estimate (i.e., prior precision, prediction errors, and environment uncertainty) can all be modulated by contextual features, such as the availability and stochasticity of immediate feedback^43^, the valence of outcomes^39^, or the number of possible choices^44–46^. As a result, modeled cognitive processes often reflect the constraints of the model more than the biologically plausible strategies that may emerge across environments^47,48^.

More complex models have incorporated dynamic updating to better approximate real-world cognition. For example, variable learning-rate models^49^, reinforcement learning models with Kalman filters^50^ or change-point models^51^ allow for trial-by-trial changes in learning rates, while Bayesian formulations such as the Hierarchical Gaussian Filter (HGF)^52^ introduce dynamic adjustments of prior and sensory precision. However, this class of models imposes normative constraints on belief updating that tie learning monotonically to uncertainty, volatility, or inferred regime changes. For instance, in Kalman filter models, the pace of learning is governed by the Kalman gain, which decreases monotonically as uncertainty about value estimates is reduced, thereby enforcing slower updating under high confidence^50^. Variable learning-rate models similarly rely on predefined mappings between learning rate and quantities such as prediction error magnitude or recent outcome variability, constraining updating to increase or decrease in a monotonic fashion as a function of surprise^49,53^. Change-point models implement a different, yet still constrained, mechanism, whereby belief updating remains suppressed within inferred stable regimes and increases abruptly only when evidence for a structural change exceeds a threshold^54,55^. More generally, computational models that are in principle compatible with the Bayesian brain hypothesis^7,56,57^ assume that arbitration between priors and evidence is expressed through ‘precision-weighting’. Within this framework, high precision of the prior (i.e., narrow distribution or high confidence in prior beliefs) downweighs new evidence and slows updating, whereas low precision of the prior (i.e., broad distribution or low confidence in prior beliefs) facilitates faster adaptation^58^. All these models preclude or severely limit the analysis of counterintuitive dynamics, such as sustained increases in updating under high belief confidence, which may emerge in clinical populations and may be meaningful when it comes to differentiating healthy from pathological cognition. Moreover, as noted above, belief updating dynamics are sensitive to environmental contexts, which can elicit fundamentally different cognitive strategies^43,59^, leading even healthy individuals to dynamically weigh priors versus evidence in unforeseen ways.

**Figure 1:**
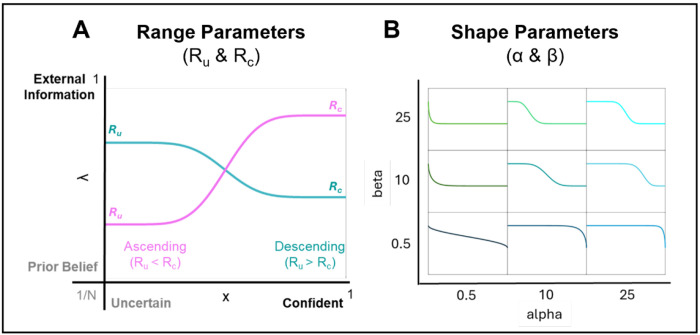
Dynamic Bayesian Model Example Parameter Curves **A)** The axes to visualize the dynamic given by the models four parameters show the relationship between reliance on external/internal information and confidence. Importantly, confidence (the x axis) is not representative of time on the task. The dynamic curves from this model show the pace of belief updating. Examples of how the range parameters influence the curve (α = 10 and β = 10 for both lines). The range parameters (Ru & Rc) represent the arbitration thresholds at maximum uncertainty (x = 1/N) and maximum confidence (x = 1). If the threshold at maximum uncertainty (Ru) is lower than the threshold at maximum confidence (Rc), the curve is ascending (magenta). If Ru > Rc, the curve is descending (teal). **B)** Examples of how α and β parameters influence the dynamic curve (Ru = 0.85 and Rc = 0.6 for all lines).

Here, we formally investigate how task environment shapes belief updating dynamics in a comprehensively-screened online sample of healthy adults (N=120) across six reversal learning environments that varied in the number of available choices, the presence of immediate feedback, and the valence of outcomes. To capture nuanced, trial-by-trial fluctuations in the arbitration between internal priors and external evidence as a function of confidence, we developed a new computational model based on Bayesian inference that allows for a broad, non-monotonic exploration of belief updating dynamics (Fig. 1).

We compared observed behavior against simulated ideal optimal performance and found that belief updating typically follows a distinctive descending arbitration curve that is near-optimal and consistent with the Bayesian brain hypothesis. However, these optimal dynamics break down when explicit reward feedback is paired with simplified choice structures or in the presence of negative outcomes. Under these conditions, healthy individuals diverge into heterogeneous, and often suboptimal, updating strategies that become apparent only through the mechanistic, dynamic Bayesian framework provided by our model.

## RESULTS

### A Novel Dynamic Bayesian Model Revealed Near-Optimal Belief Updating in Healthy Control Behavior on Standard 3-Option Tasks

**Figure 2:**
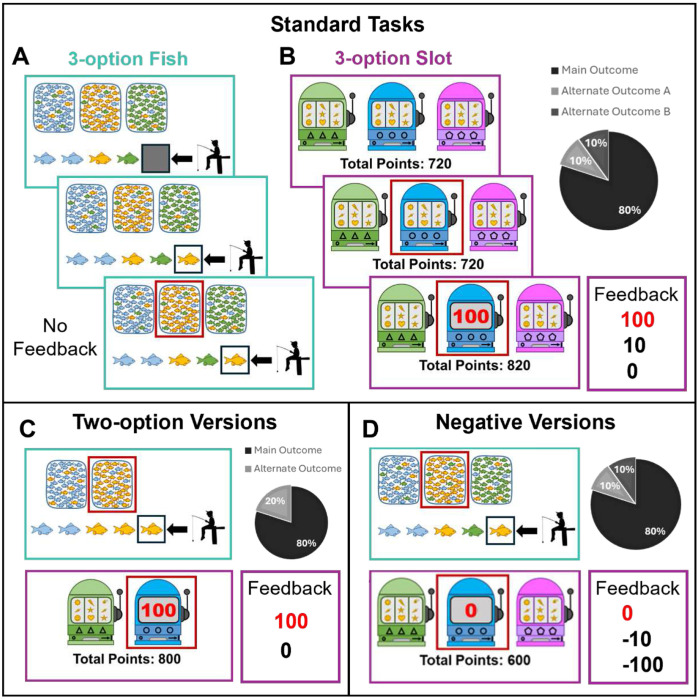
Battery of 6 Probabilistic Reversal Learning Tasks An example trial from the standard, 3-option tasks: **A)** In the fishing task, participants are asked to infer which pond the boy is fishing from based on the color of the fish caught. They are told that they win points for each correct answer. The four colored fish along the bottom reflect the outcomes of the most recent 4 trials followed by a gray box representing the start of a new trial (top slide). The fish caught on this trial is revealed as yellow (middle slide), and the participant chooses the yellow pond, as indicated by the red box (bottom slide). No feedback is given after each pond selection. Fish reveal-times for each trial varied randomly between 2.5, 3, 3.5, and 4 seconds, and pond selection was self-paced (0.5s minimum threshold). **B)** In the slot task, the possible slot options and total cumulative points are shown at the start of each trial (top slide). The participant selects a slot machine, as shown by the red box (middle slide), then the outcome is revealed, and the total points are updated (bottom slide). Reward outcomes were either 0, 10, or 100 points (bottom right box). Slot machine selection was self-paced (0.5s minimum threshold), and reward reveal-times varied randomly between 1.5, 2, and 2.5 seconds after selection. Reward presentation was fixed at 0.3 seconds on each trial. For both tasks, each option (pond/slot) had a primary output that occurred 80% of the time with the other two outcomes possible 10% of time each (pie chart). Participants played 10 blocks of 15 trials each. 8 blocks had 1 reversal point (falling between trials 5–10), 1 block had 2 reversal points, and 1 block had no reversals. C) Two-option adaptations of the Fish and Slot tasks preserved the exact structure as the standard 3-option versions, only differing in number of choices. The final slide of an example trial from the fish (top) and slot (bottom) task is shown. D) Negative adaptations of the Fish and Slot task preserved the exact structure as the standard 3-option versions, only differing in reward valence. As there is no direct reward feedback in the Fish task (top), the only difference in the negative version occurs at the start, when participants are informed that each incorrect answer results in a loss of points, and no points are earned from correct responses. In the negative version of the Slot task, participants start with 1000 points and can only lose points with 0 being the best outcome (bottom right).

We first analyzed belief updating dynamics in healthy participants performing two three-option decision-making tasks that were used as benchmarks: the *Fishing game task* (Fig. 2A), an inductive reasoning paradigm that requires inference without trial-by-trial feedback, and the *Slot machine task* (Fig. 2B), a classic three-armed bandit that provides direct reward feedback^60,61^. The comparison across tasks allowed us to assess whether our dynamic Bayesian model (DBM) generalizes across environments with distinct sources of uncertainty, whether healthy individuals behave optimally in the presence or absence of feedback, and whether the model’s dynamics offer a robust computational baseline for subsequent comparisons.

**Figure 3:**
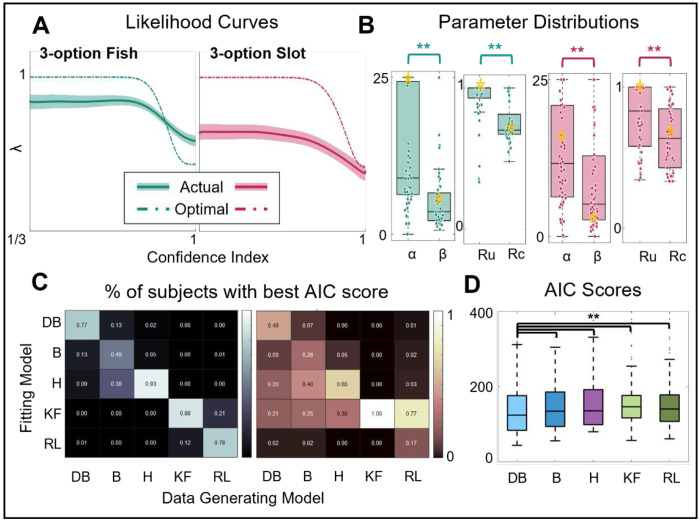
Dynamic Bayesian Model Performance on Standard Tasks **A)** Shaded error lines representing the averaged dynamic Bayesian model parameter curve for the healthy control subjects that performed the 3-option Fish task (left; n = 45) and 3-option Slot task (right; n = 55). Dotted lines represent the optimal curves for each task. Genetic algorithm optimization was used to guide the parameter search, and simulations were used to identify the set of parameters that produced the highest-performing outcomes for the simulated agent in each task. **B)** Distribution of the best fitting parameters found by the dynamic Bayesian model for HC behavior on the Fish (left) and Slot (right) tasks. Key features being α > β (Fish: cohen’s d = 0.89; p = 4.1e-07 | Slot: cohen’s d = 0.50; p = 5.4e-04) and Ru > Rc (Fish: cohen’s d = 0.54; p = 7.1e-04 | Slot: cohen’s d = 0.42; p = 3.0e-03) for the majority of subjects. These parameter features generate the particular descending curve seen in A. Yellow stars represent the optimal values for each parameter. **C)** Confusion matrices for Fish (left) and Slot (right) tasks depicting the proportion of simulated agents (N = 100) from each data-generating model (columns) for which each candidate model (rows) provided the best fit (lowest AIC score). Five models were tested: our Dynamic Bayesian (DB) model, a standard Bayesian model (B), a Heuristic model (H), a Kalman Filter Reinforcement Learning model (KF), and a standard Reinforcement Learning model (RL). The diagonal (upper left – lower right) reflects model recovery, where the fitting model matches the data-generating model. **D)** Akaike information criterion (AIC) scores for the DB model on both 3-option tasks (N =100) compared to the four other models. Paired t-tests comparing AIC scores revealed the dynamic Bayesian model significantly outperformed each of the other four models, with all comparisons remaining significant after Bonferroni correction for multiple comparisons (** : all p < .005). Black dots represent outliers.

Across both benchmark tasks, the DBM revealed a descending arbitration curve, in which the pace of belief updating decreases non-linearly as choice uncertainty decreases, or, in other words, reliance on prior beliefs increases with choice confidence (Fig. 3A). An analysis of the distribution of best-fitting parameters for each task (Fig. 3B, equations 1–3) highlights a significant relation between the parameters controlling the shape of the arbitration curve (α and β) and those controlling the range of variation of the curve itself (R_u_, for the trials with maximum uncertainty and R_c_ for the trials with maximum confidence). In particular, in both tasks, α (Fishing game 12.4 ± 8.5; Slot machine 13.3 ± 8.0) was significantly greater than β (Fishing game 5.3 ± 4.7; Slot machine 8.8 ± 8.2) (Fig. 3B, left). Similarly, R_u_ (0.91 ± 0.19; 0.78 ± 0.23) was significantly greater than R_c_ (0.75 ± 0.14; 0.64 ± 0.22) for both tasks (Fig. 3B, right). These values describe a high plateau (i.e., fast pace of update) for most trials in the task, with a sudden drop (slow pace of update) limited to high choice confidence trials.

These values are consistent with those derived in our simulations, as we used genetic algorithms to maximize average total rewards across multiple runs to estimate optimal model parameters. This method resulted in an optimal range parameter approximately equal to [R_u_ ≈ 1 (both tasks), R_c_ ≈ 0.66 (fishing game), R_c_ ≈ 0.65 (slot machine)], again characterizing a plateau with fast updates for most trials and slow update limited to high confidence trials, resulting in a descending curve. The α parameter was found to reach the upper bound of our exploration settings in the fishing game (α=25) and was approximately equal to α=16.2 in the Slot machine task. Values for the β parameter were found equal to β=5.9 and β=3.0, respectively for the fishing game and slot machine task. These parameters were tested in the range of [0,25] for α and β, and in the range [1/3, 1] for R_u_ and R_c_. Comparison between the optimal arbitration curve and the profile which emerged in healthy participants on both tasks suggests that healthy participants in both inference-based and feedback-based environments adopt near-optimal strategies.

### Dynamic Bayesian Model Performance Under Alternative Task Structures

Having established that healthy individuals rely on a normative descending arbitration strategy in the standard 3-option tasks, we next examined whether this pattern generalizes when key contextual features are altered. In particular, we tested whether belief-updating dynamics are preserved when 1) choice complexity is reduced from 3 to 2 options, as the presence of two choices has been described in the past to be associated with different computational processes and decision-making strategies^45,46^, and 2) reward valence is inverted, requiring avoidance of losses rather than pursuit of rewards. These manipulations allow us to determine whether the descending arbitration curve reflects a stable cognitive signature or one that is sensitive to specific task demands.

#### 2-Option Tasks: Descending Dynamics Persist in Inference but Degrade in Reward-Feedback Environments

**Figure 4:**
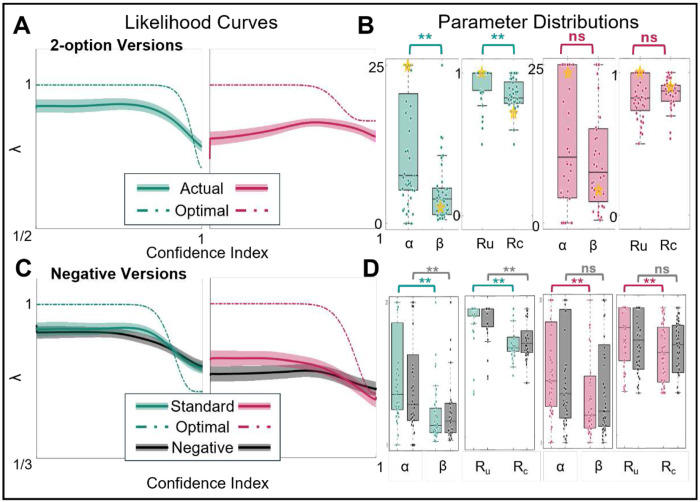
Dynamic Bayesian Model Outcomes on Alternative Tasks **A)** Averaged dynamic Bayesian model parameter curve for the healthy control subjects that performed the 2-option Fish task (left; n = 40) and 2-option Slot task (right; n = 48). Dotted lines represent the optimal curves for each task. **B)** Distribution of the best fitting parameters found by the dynamic Bayesian model for HC behavior on the 2-option Fish (left) and Slot (right) tasks. The 2-option Fish task shows the same parameter relationship: α > β and Ru > Rc. However, this parameter relationship is no longer present in the two-option Slot task. T-test comparisons revealed these differences in parameter distributions to be significant (** : p <0.05) for the Fish task and not significant (ns: p >0.05) for the Slot task. Yellow stars represent the optimal values for each parameter. **C)** Parameter curves for the healthy control subjects that performed the Negative 3-option version of the Fish task (left; n = 41) and Slot task (right; n = 43) are shown in black against the curves from the standard 3-option tasks (colored error lines). Critically, the optimal curves (dotted lines) are the same for both standard and negative versions of the 3-option tasks. **D)** Distribution of the best fitting parameters the Negative Fish (left) and Negative Slot (right) tasks shown in black compared to the standard 3-option version parameters. The Negative Fish task shows the same parameter relationship as the standard version: α > β and Ru > Rc. However, this parameter relationship is no longer present in the Negative Slot task. T-test comparisons these differences in parameter distributions to be significant (** : p <0.05) for the Negative Fish task and not significant (ns: p >0.05) for the Negative Slot task. Comparing parameter distributions from the Negative version to the standard version on both tasks revealed no statistical significance (all p >0.05).

In the 2-option Fishing game task, the group-averaged arbitration curve maintained the same descending shape observed in the standard 3-option version, closely matching the optimal model-predicted curve (Fig. 4A, left). Consistent with this, the underlying parameter structure remained intact: the average healthy participant parameter curve (α = 13.0 ± 8.7, β = 5.8 ± 6.6, R_u_ = 0.9 ± 0.1, R_c_ = 0.8 ± 0.1) again displayed the characteristic non-linear descending pattern indicative of adaptive belief updating approximating optimality with significant difference between couples of parameters in the same direction reported above: α > β and R_u_ > R_c_ (Fig. 4B, left). This indicates that when decisions must be inferred from environmental structure without explicit feedback about each choice, reducing choice complexity does not alter the normative belief-updating strategy.

In contrast, the 2-option Slot machine task did not reproduce these parameter relationships. The fitted arbitration curve (α = 12.5 ± 10.6, β = 10.4 ± 8.3, R_u_ = 0.8 ± 0.2, R_c_ = 0.9 ± 0.1) flattened and lacked the distinctive characteristics observed in the previous task versions (Fig. 4A, right). Differences between the α / β and R_u_/ R_c_ parameters did not reach statistical significance (all p > 0.05; Fig. 4B, right). Importantly, the optimal curve for the 2-option slot machine task also varied as R_u_ again converged to 1, but R_c_ was found at a higher value of ~0.9, resulting in a flatter arbitration curve (Fig. 4A). The α parameter reached optimality at 22.6, and the optimal value for the β parameter was found at 6.9. These optimization values suggest that optimality would still require the usual relations between the two couples of parameters in the DBM. Importantly, the optimal curve for the 2-option slot task was itself considerably flatter than the benchmark, 3-option task, with Rc converging to 0.9 rather than 0.65. As a result, the reported flattened arbitration curve observed in healthy participants did not constitute a significant departure from optimal performance in this environment.

#### Negative 3-option Tasks: Loss-Framed Environments Preserve Dynamics in Inference but Disrupt Dynamics in Reward-Feedback Environments

We next assessed the stability of belief-updating dynamics under negative valence conditions. Since reward maximization is theoretically identical across our tasks with different valence, optimal parameter curves are identical for benchmark positive and negative versions of each task. In the Negative Fishing game task, the fitted arbitration curve again closely approximated the optimal descending shape (Fig. 4C, left), and the signature parameter relationships (α > β and R_u_ > R_c_) remained significant (Fig. 4D, left). Parameter distributions did not differ significantly from those obtained in the positive version, indicating that inference-based updating is robust to changes in reward framing.

In contrast, participant performance on the Negative Slot machine task showed a breakdown of the optimal arbitration curve that resembled the one described for the 2-option version of the task. The average arbitration curve flattened, considerably diverging from the optimal (Fig. 4C, right). The parameter relationships described for the benchmark positive version of the task were absent (α ≈ β and R_u_ ≈ R_c_; all p > 0.05; Fig. 4D, right). This suggests that explicit feedback delivered in a negative reward frame can alter how healthy individuals arbitrate between priors and evidence as a function of confidence. Critically, unlike the 2-option Slot machine task, the flattened curve in the negative context marks a significant deviation from optimality. Thus, even in healthy populations, loss-framed environments induce suboptimal belief-updating strategies.

### Divergent Arbitration Strategies Underlie Flattened Curves in Alternative Slot machine Tasks, with Suboptimality Emerging Only in the Negative Condition

**Figure 5:**
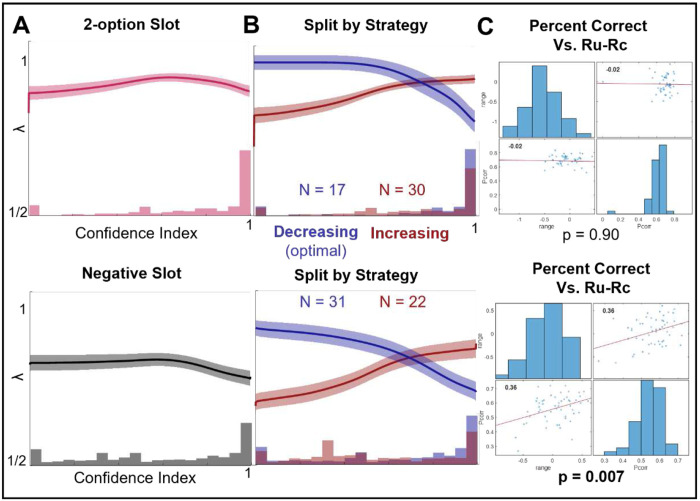
Divergent Arbitration Strategies in Alternative Slot Tasks Reveal Strategy-Dependent Performance Only in the Negative Condition **A)** Averaged dynamic Bayesian model parameter curve for the healthy control subjects that performed the 2-option Slot task (top; n = 48) and Negative 3-option Slot task (bottom; n = 53). The histogram at the bottom of the plot reflects the proportion of trials corresponding to each confidence bin. **B)** Subjects were split by arbitration strategies: descending curves (blue; Ru − Rc > 0) and ascending curves (red; Ru − Rc < 0). **C)** Correlation panels show the relationship between arbitration curve range (Ru-Rc) and task performance. Top: The 2-option slot task shows no association between strategy and performance (ns; p>0.05). Bottom: The negative slot task shows a strong positive correlation, with descending strategies predicting higher performance (p < 0.05).

The group-level comparison across versions of the Slot machine task highlighted significant differences in arbitration curves, with marked suboptimality in the Negative version of the task. To understand the origin of these flattened curves, we next examined whether this pattern reflected a single, uniform updating strategy or the aggregation of heterogeneous strategies across individuals. Across both tasks, we found a bimodal distribution of fitted arbitration ranges (ΔR: R_u_ − R_c_) revealing two distinct subgroups: participants who used a descending (optimal) arbitration and those who used ascending curves (Fig. 5) When these subgroups were plotted separately, the opposing profiles became clear. The flat group-level curves were not indicative of a shared updating policy but instead reflected the average of two diverging behavioral policies (Fig. 5A–B), where one group followed a canonical arbitration curve and the other was characterized by an inversion, with decreased updating in high choice uncertainty trials and increased updating in high choice confidence trials.

Despite this heterogeneity, in the 2-option Slot machine task, there was no relationship between ΔR and task performance (p > 0.05; Fig. 5C, top), indicating that both descending and ascending strategies yielded comparable outcomes in this simplified reward environment; both groups showed only minor performance differences in comparison with the ideal optimal. In contrast, in the Negative Slot machine task, there was a strong positive association between ΔR and performance (Fig. 5C, bottom): participants who relied on descending (optimal) strategies performed significantly better than those who adopted ascending curves, which proved markedly suboptimal. Notably, 22 of the 53 healthy participants used this suboptimal ascending strategy despite its clear performance cost. Additional demographic comparisons revealed no significant differences between “optimal” and “sub-optimal” groups in sex, age, education, income, or social deprivation index. These findings demonstrate the utility of the dynamic Bayesian model in uncovering latent strategy heterogeneity and identify when deviations from optimal arbitration meaningfully impact behavior.

### Model Comparison

Lastly, we assessed whether the DB model provided a better mechanistic account of behavior than four alternative models that formalize updating through fixed learning rates or monotonic assumptions: a standard Bayesian model, a heuristic choice model, a Kalman-filter reinforcement learning model, and a standard reinforcement learning model. Model-recovery analyses demonstrated strong recoverability of the DB model in both task environments, with simulated data generated by the DB model most often fit best by the DB model itself and minimal misidentification by competing models (Fig. 4C). Finally, we used the Akaike information criterion (AIC) score comparison to assess the DB model’s performance and ability to generalize across task contexts. AIC scores were computed from both benchmark tasks, ensuring that models were evaluated on their capacity to capture behavior consistently in both inference and reward-feedback environments. This combined analysis showed that our DBM significantly outperformed all four competing models (paired t-tests, Bonferroni-corrected; Fig. 4D). By evaluating model performance jointly across tasks rather than in isolation, this analysis validates the use of this more complex model, which offers the most robust and generalizable account of belief updating across distinct forms of uncertainty.

## DISCUSSION

This investigation addressed two interrelated challenges in the computational study of how humans arbitrate between evidence and prior beliefs: overcoming common structural constraints in existing models and capturing the full range of task-specific dynamics that emerge in human decision-making. We developed a dynamic Bayesian model capable of capturing non-monotonic, trial-by-trial fluctuations in the arbitration between priors and evidence and applied it across six reversal learning environments that varied in feedback structure, choice complexity, and outcome valence. Across four of the six tasks tested (the 3-option Fishing game and 3-option Slot machine tasks with positive rewards, the 2-option Fishing game, and the negative Fishing game tasks) we consistently observed behavior dynamics that approximate an optimal descending and non-linear arbitration curve. This pattern, characterized by high reliance on current evidence under choice uncertainty (R_u_ =1) and a significant reduction in this reliance as choice confidence grows (R_c_ = 0.7), in favor of prior beliefs, reflects a computationally optimal strategy for adaptive belief updating in probabilistic environments subject to unpredictable reversals. Notably, these empirical findings mirrored our simulations, where the optimization process achieved using genetic algorithms consistently identified similar parameter configurations. These dynamics match classic interpretation of brain computations that have been used to provide powerful explanatory models for healthy cognition and psychiatric dysfunction alike^62,63^.

The Bayesian brain hypothesis posits that perception and decision-making arise from the integration of prior beliefs and sensory evidence weighted by their relative precision. Within this framework, belief updating is expected to decrease as posterior precision increases, because highly confident beliefs are less susceptible to revision by new observations^64^. Similarly, the accounts based on reinforcement-learning algorithms assume that in volatile environments with occasional reversals, optimal agents increase learning when outcomes are highly surprising (i.e., unexpected or uncertain) and reduce learning once a reliable value representation has been established^5^. At the neural level, these computations have been classically framed in terms of attractor dynamics: healthy systems rely on neuromodulators to dynamically shift between stability, where existing configurations resist perturbation, and instability, where state transitions are easily triggered by new sensory inputs^65–67^. The descending arbitration curve we observed, with high evidence weighing under high choice uncertainty and increasing reliance on priors under high choice confidence, is consistent with these computational, algorithmic and implementational hypotheses, as adaptive systems transition smoothly between these sources of information. The question, then, is can task environments disrupt the emergence of this adaptive dynamic?

For the slot machine task, we found that a reduction in choice complexity (from three to two options) was sufficient to observe a divergence from this otherwise robust dynamic. Here, the arbitration curve flattened, with R_c_ values converging toward R_u_, indicating an apparent fixed arbitration between new information and priors, unaffected by changes in choice confidence. This finding can be understood in the context of our simulations, as the genetic algorithm identified a reduced difference between R_c_ and R_u_ (~0.10), in comparison with previous tasks [0.23-0.35], indicating that while a distinction was present, it was far less pronounced. These results suggest that binary reward-feedback environments impose little need for a strategic shift from evidence-driven to belief-driven updating. Consequently, participants, on average, did not reliably adopt descending curves; many used ascending strategies, yet performed equivalently well. Crucially, these subtle dynamics, and the heterogeneity of strategies producing them, were only detectable using our dynamic Bayesian model, as traditional reinforcement learning or heuristic analyses would obscure them entirely. This finding highlights a key limitation of the two-armed bandit paradigm, one of the most widely used tasks in computational psychiatry^14^. In binary choice environments with explicit, immediate reward feedback, belief updating may not require a genuine arbitration between priors and incoming evidence. Participants may either rely on shallow but effective policies that do not require arbitration between priors and evidence or deeper belief-updating computations, or their arbitration dynamics may be obscured because, in a bandit task with reversals, information about both choices can be inferred regardless of which option is chosen. In either case, the structure of the task makes it difficult to determine whether apparent stability in behavior reflects true confidence-weighted updating or simply a reduced need for arbitration, thereby limiting interpretability. This contrasts with the binary Fishing task, where choices must be inferred without direct feedback, and where descending dynamics remain intact.

By contrast, our findings in relation to the negative three-option slot machine task, where outcomes were framed as losses rather than rewards, are more challenging to explain. In this case, healthy participants again diverged into subgroups exhibiting descending and ascending arbitration strategies; however, the divergence was clearly suboptimal as participants relying on ascending arbitration strategies showed significantly poorer performance and lower overall rewards. Because we could not identify any demographic or survey-based measure associated with these behavioral differences, we can only speculate about the mechanisms underlying this separation into two distinct phenotypes. For instance, negative outcomes may be interpreted by some participants as threatening or more risk-inducing, in comparison with the standard reward-only version of the task, despite the identical probability structure and ideal optimal choice strategy. Under these conditions, avoidance of losses or risk aversion may promote the emergence of suboptimal strategies in a significant subset of participants, including in healthy controls. These findings suggest that computational models of commonly used negative paradigms (e.g., see^68,69^) need to be designed to capture latent heterogeneity in choice behavior and to identify contexts in which such heterogeneity becomes behaviorally meaningful, including, potentially, clinically heterogenous cohorts.

One study limitation should be noted. Although data were collected via Prolific’s vetted participant pool, we cannot fully rule out the presence of large language model-driven bots, which recent work suggests can evade standard quality controls^70^. However, Prolific appears to be less affected by this issue than other platforms, data collection began in late 2024, and we did not provide any information about required demographics or disorders of interest, making it less likely that an agent might have been instructed to respond. Additionally, one item required active interaction with an external website to retrieve one’s own social deprivation index, a task beyond the capability of most agents at the time of data collection.

In conclusion, our study indicates that choice complexity and feedback structure shape the cognitive computations engaged during belief updating, with important implications for task design and data interpretation. Future work will explore how these and other task features (e.g., reward magnitude, volatility) interact to shape belief updating dynamics in clinical populations with known decision-making impairments. For instance, in OCD, mixed patterns of both accelerated and slowed belief updating have been reported across studies relying on armed bandits with reversals^35,71^. If task context can produce heterogeneous (and even suboptimal) updating strategies in healthy individuals, then apparent contradictions within a disorder may partly reflect natural responses to differences in task structure rather than inconsistencies in the underlying pathology. Our dynamic Bayesian model effectively characterized the dynamics of belief updating across probabilistic tasks, revealing context-dependent behaviors and identifying when and how adaptive strategies break down, offering promising avenues for identifying distinct computational mechanisms that could differentiate psychiatric patient populations.

## MATERIALS AND METHODS

### Participant Screening and Recruitment

#### Recruitment Platform and Initial Screening

Participants were recruited through the online crowdsourcing platform Prolific (Prolific.com) between May 2024 and October 2025. Eligibility criteria included fluency in English, a minimum age of 18, and residency in the United States. Approximately 1500 individuals completed an initial screening survey taking approx. 5 minutes to complete. This survey gathered demographic data and general mental health information, with particular attention to preexisting psychiatric conditions. Participants were informed that, if selected, they would be invited to a longer study lasting approximately 1 hour 15 minutes, including a battery of surveys and tasks. All participants provided electronic informed consent prior to any data collection.

#### Psychiatric Questionnaires and Formation of Healthy Control Sample

**Figure 6:**
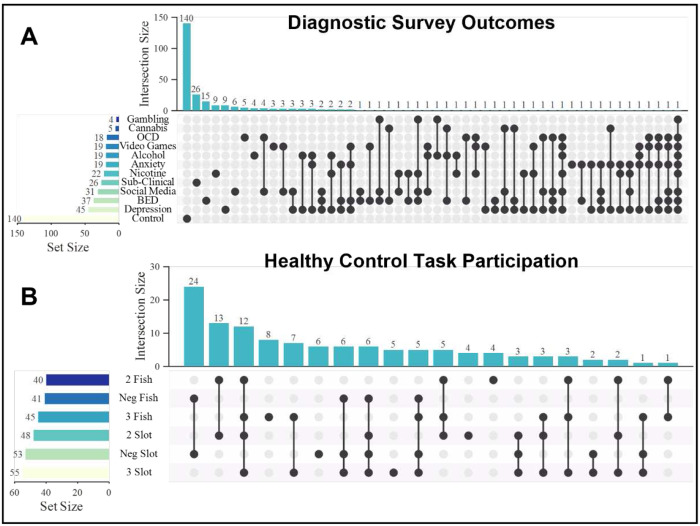
Participant Survey and Task Battery Outcomes **A)** All pre-screened participants (N = 276) were prompted to complete a thorough psychiatric survey. This upset plot visualizes participant diagnostic outcomes after completing the 10-questionnaire survey. Vertical bars represent the number of subjects meeting criteria for each diagnostic profile (indicated by filled in black dots below). Horizontal bars show the number of subjects meeting criteria for each category, irrespective of intersection (co-occurring diagnostics). **B)** All verified healthy control participants (N = 140) were invited to participate in a battery of 6 decision-making tasks. This upset plot visualizes participants who completed each task. Vertical bars represent the number of subjects who completed each combination of tasks (indicated by filled in black dots below). Horizontal bars show the number of subjects who completed each task, irrespective of intersection.

Individuals who reported no preexisting psychiatric health conditions or endorsed specific disorders of interest and passed three attention checks on the initial screening were invited to participate in the full study. Here, we focus only on results from healthy controls in the pool as efforts to expand our pool of disorders of interest are ongoing. A total of 276 pre-screened participants completed the full study, which consisted of a battery of self-report psychiatric questionnaires (taking approximately 45 minutes; Table 1) and up to six tasks (approximately 10 minutes each; Table 2). The surveys included well-established self-assessments for 10 common psychiatric domains that allowed us to identify healthy individuals based on score cutoffs described in the literature. From the initial pool of 276 respondents, 140 subjects matched our criteria (Fig. 6A). Further exclusion criteria were applied based on task performance (see below), yielding a total of N = 120 subjects. Only individuals who qualified as healthy on all screening measures and passed task performance and attention checks were included in the present study. This study was reviewed and approved by the Institutional Review Board at the Icahn School of Medicine at Mount Sinai (IRB-18-01301) and was determined to meet criteria for minimal risk research, as the study involved no collection or use of identifiable protected health information.

Access to the tasks was characterized by counterbalanced order and staggered invitations over the course of up to four months from completion of the surveys. Participants could decide to participate in any or all of the six decision-making tasks in the study, resulting in a variable number of participants per task (Fig. 6B). To be included in the final behavioral analysis on each task, participants had to pass all attention checks and basic task performance criteria. Across all tasks, we excluded participants whose choices reflected simple heuristic strategies. On the Fishing game tasks, this simple heuristic is known as a “color-following” strategy, which is defined as selecting the pond which exactly matches the fish color presented on each trial. Anyone who had >95% color-following trials were excluded on this basis. Similarly, on the Slot machine tasks, the simple heuristic is known as a “win-stay-lose-shift” (WSLS) strategy, which is defined as selecting the same slot machine following a winning outcome (i.e., 100 pts in the standard task and 0 pts in the negative version), and switching after every losing outcome. Participants who had >95% WSLS trials were excluded. Each participant received monetary compensation consistent with Prolific’s recommended rate, plus task-related performance bonuses(approximately $20/hour base compensation for their time, plus an approximately $8 per performance-based bonus per task).

### Behavioral Tasks

We analyzed the dynamics of belief updating in non-static environments using probabilistic learning tasks with and without immediate feedback ^72,73^. Each paradigm required participants to infer hidden state changes based on sequential probabilistic evidence. The general structure (number of reversals, number of trials, block organization) was kept stable across tasks. To specifically investigate the influence of choice complexity on belief update dynamics, we tested the same task structures using both three- and two-option versions^44–46^.

In the Fishing Task (Fig. 2A) participants were told a boy is catching fish from one of three possible ponds, each depicted on screen as associated with a dominant fish color (blue, yellow or green), with an 80% probability of yielding its primary fish color, and 10% probability for each of the two remaining “rare” colors. One fish is presented in each trial to the participants, who are asked to infer from which pond it was caught. Participants are told they win points each time the correct pond is selected. The task consisted of 10 blocks of 15 trials each. Within each block, the pond from which the boy was fishing could change 0, 1, or 2 times, creating reversal points (one reversal on average per block, with only one block with 0 reversals and one block with 2 reversals). Participants were not explicitly informed when a reversal occurred and did not receive immediate feedback on the accuracy of their choices, but they were told the boy fishing in the game “may or may not” change the pond during each block of trials. Despite the lack of trial-by-trial information, participants were instructed to maximize their total hidden points, as a final reward would be calculated and displayed at the end of the task, and each correct guess would be converted into 100 points.

The Slot Machine Task (Fig. 2B) relies on a similar structure to the Fishing Task in terms of number of trials, blocks, and reversals (15 trials per each of 10 blocks, one reversal on average per block, with only one block with 0 reversals and one block with 2 reversals) but introduced stochastic explicit rewards after each choice as an outcome measure. As in classic multi-armed bandit tasks, participants were presented with three slot machines, each predominantly yielding one of three possible reward values: 0, 10, or 100 points. Each slot produced its primary reward 80% of the time, while the alternative rewards occurred with 10% probability each. Participants were instructed to maximize their total points, thereby incentivizing them to identify the slot machine that dispensed a primary reward of 100 points.

To examine how the number of choice options affects belief updating, we also implemented a simplified version of the Fishing and Slot machine Tasks with only two options (Fig. 2C). In these versions, the dominant fish color for each pond and reward outcome for each slot remained at 80% probability, while the alternative color and outcome appeared 20% of the time. The trial, block and reversal structure of the original 3-option versions remained unchanged.

To examine how negative valence affects belief updating, we implemented a negative version of the 3-option Fishing and Slot machine tasks (Fig. 2D). In the negative Fishing task, the only difference from the standard version was in the initial framing of the task – participants were told that they lose points for each incorrect answer, rather than gaining points for correct responses. In the negative Slot machine task, participants start each block with 1000 points and can only lose points, with 0 being the best outcome.

For all tasks, participants were informed that a final bonus based on performance would be calculated at the end of the task based on a randomly extracted block, and they would earn $1 per 100 points.

### Computational Modeling

#### Dynamic Bayesian Model

We developed a dynamic Bayesian update model to capture how participants change, on a trial-by-trial basis, their reliance on priors vs. current evidence when updating beliefs. The model was developed with the aim to investigate shifting decision-making policies both in the presence (Slot machine task) and in the absence (Fishing task) of immediate rewards, with minimal computational adjustments. The model’s approach draws on standard Bayesian observer principles: prior beliefs are considered when computing new observations (i.e., a colored fish or a reward after a slot machine choice), to form posterior beliefs, following standard Bayesian update^7^:

(1) $$P{\left({\mathrm{pond}}_{j}\right)}_{t}\propto \lambda_{1}P{\left({\mathrm{pond}}_{j}\right)}_{t-1}$$

(2) $$P{\left({\mathrm{SlotMachine}}_{j}\right)}_{t}\propto \lambda_{2}P{\left({\mathrm{SlotMachine}}_{j}\right)}_{t-1}$$

Where $\lambda_{1}=P\left({\mathrm{pond}}_{j}\right)$ or the likelihood to catch a fish of a determinate color (c) from each of the possible ponds (j), and $\lambda_{2}=P\left({\mathrm{SlotMachine}}_{j}\right)$ or the likelihood to gain a reward of 100 points, if the slot machine j is chosen. In these models, the value of the likelihood λ is usually assumed to be fixed (per subject, per task), and a high value of λ (≈1) is optimal in environments that are assumed to be quasi-deterministic, where the belief updating process can rely significantly more on current evidence rather than priors, with associated fast updates. Conversely, low values of λ (i.e., in our task structures, ≈1/n, where n equals the number of available fish colors or outcomes) are optimal in highly stochastic environments, which require a high reliance on prior beliefs rather than current evidence, ignoring noise and resulting in slow updates.

The key innovation of our version of this classic model is in the possibility to dynamically vary the value of λ on a trial-by-trial basis in either direction. We used a transfer function to create a “dynamic arbitration” and investigate how reliance on prior beliefs vs. current evidence (and associated pace of belief updating) varies as a function of the priors, as follows:

(3) $$\left\{\begin{array}{l} \lambda_{t}=R_{u}-B\left(\max\left(P{\left(.\right)}_{t-1}\right),\;\alpha ,\beta \right)*\mathrm{abs}\left(R_{u}-R_{c}\right),\;R_{u}>R_{c}\\ \lambda_{t}=R_{u}+B\left(\max\left(P{\left(.\right)}_{t-1}\right),\;\alpha ,\beta \right)*\mathrm{abs}\left(R_{u}-R_{c}\right),\;R_{u}\leq R_{c} \end{array}\right.$$

Where $\left[\left(P(.{)}_{t-1}\right)\right]$ indicates the maximum value of the vector of the prior, $R_{u}$ and $R_{c}$ are free parameters within $\left[\frac{1}{n},1\right]$ and respectively represent the value of $\lambda_{t}$ for $\max\left(P(.{)}_{t-1}\right)=1/n$ (maximum prior uncertainty or low precision) and $\max\left(P(.{)}_{t-1}\right)=1$ (maximum prior confidence or high precision), therefore determining the “range” of variation of the arbitration. Finally, B(.) indicates the incomplete Beta function, controlled by the free parameters α and β. This transfer function is used to allow for the free exploration of different dynamics (e.g., linear, sigmoid, hyperbolic, etc.) controlling the arbitration between prior and evidence, so to determine the dynamics of the pace of updates, as a function of the maximum value of the prior distribution, also providing a clear way to visualize potential changes in dynamics across tasks (Fig. 3). Crucially, parameter optimization can result in a broad spectrum of linear and non-linear dynamics, including monotonically increasing and decreasing curves, where the weight of either priors or evidence (and associated pace of belief updating) can increase or decrease as a function of the maximum value of the prior.

#### Model Comparison

We compared our new model against four alternative benchmark models commonly used to describe decision-making tasks with reversals. First, a standard Bayesian observer^74–77^ used a fixed likelihood estimated per subject, for all trials in a task (*λ*_1_ *and* *λ*_2_ in equations 1 and 2), rather than a dynamically adjusting arbitration parameter. Second, a standard reinforcement learning (RL) model^5^, based on Rescorla-Wagner prediction error estimates, to update subject-specific values of available choices. Third, we included a RL model with a Kalman filter^50^ to capture a more dynamic form of reinforcement learning in which the learning rate varies trial-by-trial as a function of the estimated reward uncertainty. Finally, a heuristic model was developed based on simplified decision rules.

#### Standard Bayesian Model

The standard Bayesian observer relies on categorical evidence (e.g., fish color/slot outcome) to update beliefs about which option is correct. As in equations (1) and (2), prior beliefs about each option are multiplied by the likelihood of observing the outcome under that option, yielding updated posterior beliefs on each trial:

(4) $$P_{t}(i)=\frac{\lambda_{i}(o)*\;P_{t-1}(i)}{\sum_{j}\lambda_{j}(o)*\;P_{t-1}(j)}$$

Here, $P_{t}(i)$ is the posterior belief that option *i* is correct at trial $t,P_{t-1}(i)$ is the prior belief from the previous trial, and $\lambda_{i}(o)$ is the likelihood of observing *o* if option *i* is correct. The denominator represents the total probability of the observed outcome across all possible options and ensures that the posterior probabilities sum to 1. In this standard formulation, *λ* is fixed for each subject and task. Thus, the model assumes that the observer applies the same likelihood throughout the task, reflecting a consistent reliance on evidence relative to priors. This captures the canonical Bayesian update and does not allow the arbitration between prior beliefs and evidence to vary dynamically across trials.

#### Rescorla-Wagner Reinforcement Learning Model

In this RL model, a value (*Q*) is estimated for each option *i*. In the slot machine task, after each trial, the chosen option’s value (*Q*_*i*_) is updated based on the difference between the observed reward at the trial (*r*_*t*_) and the expected reward associated with the choice option *Q*_*t*−1_ (*i*), scaled by a learning rate (*α*), as follows:

(5) $$Q_{t}(i)=Q_{t-1}(i)+\alpha \left[r_{t}-Q_{t-1}(i)\right]$$

For the non-chosen options, values were updated toward the average reward of the unselected choices, scaled by the same learning rate, to reflect learning about forgone alternatives. The updated values are then converted into choice probabilities using a softmax function, where a parameter controlling the temperature (τ) regulates a signal-to-noise ratio to determine the probability of selecting the higher-valued option rather than exploring other options. For all 3-option tasks, τ was fixed at 10, the value approximated as best fitting behavior when treated as a free parameter. On the 2-option tasks, preliminary model fitting revealed a value of τ ≈20 was required to improve model performance.

The RL framework was naturally suited to the slot machine task, which provided explicit numeric rewards (0, 10, 100 points) on each trial. However, the fishing game was designed to test decision making in the absence of immediate reward feedback. To apply the RL model consistently across both tasks, we treated the colored fish presented on each trial as either a ‘win’ (100-point reward) if the new fish color matched the previously chosen pond, or a ‘loss’ (0-point reward) otherwise. This translation preserved the basic RL principle – updating a value estimate based on the difference between observed and expected outcomes – while acknowledging that, in the fishing game, the ‘reward’ is inferred from color matching rather than being visibly quantified.

#### Kalman Filter Reinforcement Learning Model

To test an alternative dynamic approach, we integrated a Kalman filter into the standard RL framework, allowing the model to adaptively adjust its learning rate based on trial-by-trial uncertainty in the estimated values. In this model, each option’s estimated value *Q*_*t*_(*i*) is treated as a latent state with associated uncertainty *σ*_*t*_(*i*). Whenever a reward is observed (in the slot machine task) or inferred (in the fishing game), the model computes a Kalman gain *K_t_*(*i*), which depends on how uncertain it is about the current estimate (*σ*_*t*−1_(*i*)) and the assumed observation noise *ν*. The gain effectively becomes an “adaptive learning rate” – if uncertainty is high, the learning rate increases, whereas low uncertainty dampens the effect of the prediction error:

(6) $$K_{t}(i)=\frac{\sigma_{t-1}(i)}{\sigma_{t-1}(i)+v}$$

(7) $$Q_{t}(i)=Q_{t-1}(i)+K_{t}(i)\left[r_{t}-Q_{t-1}(i)\right]$$

(8) $$\sigma_{t}(i)=\left(1-K_{t}(i)\right)\sigma_{t-1}(i)+v$$

The same considerations concerning decision via softmax function and τ values described for the RW model apply to the Kalman filter RL model to ensure comparability.

#### Heuristic Model

This simplified model is based on a single parameter *h*, used to adjust the probability of choosing each option by adapting a simple “win-stay, lose-shift” rule. After each trial, if the chosen option yields a win (e.g. a matching fish color or a 100 point outcome), that options probability is increased by *h*, and the probability to select the remaining options is equally adjusted by subtracting the complementary value, divided by the number of remaining choices. The opposite mechanism is true, in case of a loss.


(9) $$P_{t}(\mathrm{chosen})=(1,P_{t-1}(c)+h)$$

(10) $$P_{t}(\mathrm{other})=\max(0,P_{t-1}(o)-\frac{h}{\left(N-1\right)})$$

As described in our previous model-based analysis of choice behavior and model comparison with similar tasks^43^, after the update and the calculation of the log likelihood for parameter optimization, we implemented a floor minimum P(0.05) for all choices, across Bayesian models and heuristic model. The vector of probabilities is then normalized to become the new prior. This is meant to account for a residual level of exploratory behavior, without biasing the process of parameter optimization.

### Simulations of Optimal Behavior and Model Recovery

To identify the parameter settings that produced optimal performance for each task environment, we employed a genetic algorithm (GA) optimization procedure. For each task, we defined a fitness function that evaluated model performance as the negative prediction error between simulated and ideal choice behavior (i.e., maximizing average reward and minimizing error relative to the generative structure of the task). Model parameters subject to optimization included R_u_, R_c_, and the shape parameters alpha and beta of the transfer function (equation 3). The GA was initialized with a population of candidate parameter sets sampled uniformly across the allowable parameter space. Across successive generations, parameter sets with higher fitness values were preferentially retained with random mutations introduced to maintain diversity and avoid local minima. The procedure terminated when convergence criteria were met (no improvement in fitness over 50 generations) or after 1,000 generations, whichever occurred first. This approach ensured a broad and efficient search of the parameter space, yielding parameter values that approximated optimal belief-updating dynamics for each task structure.

To assess model performance on each task, we constructed a confusion matrix in which a simulated dataset (N=100 agents) was generated under each model and resulting behavior was fit with all candidate models. The proportion of simulated agents for which each candidate model provided the best fit (lowest AIC score) was tabulated. Simulated agents were generated using biologically plausible parameter values derived from the best-fitting parameters estimated on real participant data, with added +/−10% variance to expand the sample to 100 agents.

## Figures and Tables

**Table 1:** Diagnostic screening tools

Diagnostic	Screening Tool	Score Range	HC Threshold	Diagnostic Threshold
**Anxiety**	GAD-7	0-21	<5	>9
**Depression**	CESD-10	0-30	<10	>9
**OCD**	OCI-R	0-72	<21	>20
**Alcohol**	AUDIT	0-40	<8	>13
**Nicotine**	FTCD	0-10	<1	>=3
**Cannabis**	CUDIT	0-32	<1	>11
**Binge Eating**	EDEQ*	0+	<4	>=4
**Gambling**	GSAS	0-48	<9	>20
**Video Games**	VGAQ	1-6	<=2	>2
**Social Media**	SMAQ	1-6	<=2	>2

Participants were screened for the listed 10 psychiatric diagnostics. Total scores were calculated according to existing literature for each screening tool. VGAQ and SMAQ scores and thresholds reflect the average score/question.

* Binge Eating was determined based on participant responses to a single question on the EDEQ: ede_q_6_14.

**Table 2:** Healthy Adult Task Participation

Task	Total	Final
**3 Fish**	56	**45**
**2 Fish**	46	**40**
**Neg Fish**	53	**41**
**3 Slot**	55	**55**
**2 Slot**	49	**48**
**Neg Slot**	56	**53**

All verified healthy participants (N = 140) were invited to participate in a battery of 6 decision-making tasks. This table displays the total number of HC participants who completed each task, and the final number of those participants who then met basic attention and task performance criteria (see methods). The Final HC subjects (bold) are those used in the analyses henceforth.

## Data Availability

All data required to replicate the findings, including choice behavior, surveys, computational models and simulation pipelines, and all related dictionaries will be made available for download in a github.com repository upon acceptance of the manuscript.
